# The heat flow for the full bosonic string

**DOI:** 10.1007/s10455-016-9514-4

**Published:** 2016-05-31

**Authors:** Volker Branding

**Affiliations:** Institut für diskrete Mathematik und Geometrie, TU Wien, Wiedner Hauptstrasse 8-10, 1040 Vienna, Austria

**Keywords:** Harmonic maps with scalar and two-form potential, Full bosonic string, Heat flow, 58E20, 35K55, 53C08, 53C80

## Abstract

We study harmonic maps from surfaces coupled to a scalar and a two-form potential, which arise as critical points of the action of the full bosonic string. We investigate several analytic and geometric properties of these maps and prove an existence result by the heat-flow method.

## Introduction and results

Harmonic maps between Riemannian manifolds are one of the most studied variational problems in differential geometry. Under the assumption that the target manifold has non-positive curvature Eells and Sampson established their famous existence result for harmonic maps making use of the heat-flow method [10]. Moreover, in the case that the domain is two-dimensional harmonic maps belong to the class of conformally invariant variational problems yielding a rich structure.

However, harmonic maps from surfaces also have a dual life in theoretical physics. More precisely, they arise as the *Polyakov action* in bosonic string theory. The full action for the bosonic string contains two additional terms, one of them being the pullback of a two-form from the target and the other one being a scalar potential. It is the aim of this article to study the full action of the bosonic string as a geometric variational problem.

There are already several mathematical results available for parts of the energy functional of the full bosonic string: in [11] the authors consider the harmonic map energy together with a scalar potential. The critical points of this energy functional are called *harmonic maps with potential*. One of the main results in that reference is that depending on the choice of potential, the qualitative behavior of harmonic maps with potential differs from the one of harmonic maps. There are several results available that characterize the properties of harmonic maps with potential: this includes gradient estimates [6] and Liouville theorems [7] for harmonic maps with potential from complete manifolds. In [8] an existence result for harmonic maps with potential from compact Riemannian manifolds with boundary is obtained, where it is assumed that the image of the map lies inside a convex ball. An existence result for harmonic maps with potential to a target with negative curvature was obtained in [12] by the heat-flow method. This result has been extended to the case of a domain manifold with boundary in [9]. Harmonic maps from surfaces coupled to a two-form potential have also been studied in the mathematical literature since they give rise to the *prescribed mean curvature equation*. Existence results via the heat flow for the prescribed mean curvature equation have been obtained for a flat target in [23] and for a three-dimensional target with negative curvature in [27].

In theoretical physics the two-form potential is interpreted as giving rise to an external magnetic field.

Harmonic maps coupled to a two-form potential and spinor fields instead of a scalar potential have been studied in [4].

We will call the critical points of the full bosonic string action *harmonic maps with scalar and two-form potential* and we will generalize several results already obtained for harmonic maps and harmonic maps with potential. Moreover, we will point out new phenomena that arise from the two-form potential.

This article is organized as follows. In Sect. 2, we analyze the energy functional of the full bosonic string and derive its critical points. In Sect. 3, we study analytic and geometric aspects of the critical points. In the last section, we derive an existence result via the heat-flow method for both compact and non-compact target manifolds.

## The full bosonic string action

Throughout this article (*M*, *h*) is a Riemannian surface without boundary, we will mostly assume that *M* is compact, and (*N*, *g*) a closed, oriented Riemannian manifold of dimension $\dim N\ge 3$. For a map $\phi :M\rightarrow N$ we consider the square of its differential giving rise to the usual harmonic energy. Let *B* be a two-form on *N*, which we pull back by the map $\phi$. In addition, let $V:N\rightarrow \mathbb {R}$ be a scalar function, which we mostly assume to be smooth. By *R* we denote the scalar curvature of the domain *M*.

In the physical literature the full action for the bosonic string is given by

2.1 $$\begin{aligned} E(\phi )=\int _M\left( \frac{1}{2}|{\mathrm{d}}\phi |^2+\phi ^*B+RV(\phi )\right) \mathrm{d}M, \end{aligned}$$

see for example [22, p. 108]. Due to the uniformization theorem we can assume that the scalar curvature *R* on the domain *M* is constant. In string theory, the potential $V(\phi )$ is usually referred to as *dilaton field*.

For the sake of completeness, we want to mention that in theoretical physics one also defines an action functional that locally has the form (2.1) but has a different global structure. More precisely, one replaces the two-form contribution in (2.1) by an object that locally looks like a two-form, but forms a more general object from the global point of view. One then studies the *U*(1)-valued functional

2.2 $$\begin{aligned} \exp (iE(\phi )):=\exp \left( i\int _M\left( \frac{1}{2}|{\mathrm{d}}\phi |^2+RV(\phi )\right) {\mathrm{d}}M\right) \cdot {\text {hol}}(\phi ), \end{aligned}$$

where ${\text {hol}}(\phi )$ denotes the holonomy of the gerbe ${\mathcal G}$ along the map $\phi$. By the three-form $\Omega$ we denote the globally defined curvature associated to the gerbe ${\mathcal G}$. For a mathematical introduction to gerbes and surface holonomy see [13]. This gives rise to the so-called *B-field action* in string theory, which is defined as

$$\begin{aligned} E_B(\phi ):=-i\log ({\text {hol}}(\phi )) \end{aligned}$$

such that $${\text {hol}}(\phi )=\exp (iE_B(\phi ))$$.

In the case that the gerbe ${\mathcal G}$ is trivial, which corresponds to the fact that the three-form $\Omega$ is exact, we can define the *B-field action* as

$$\begin{aligned} E_B(\phi ):=\int _M\phi ^*B{\mathrm{d}}M \end{aligned}$$

leading to the functional (2.1). From an analytical point of view the *U*(1)-valued functional (2.2) is more difficult, and we will restrict ourselves to the functional (2.1).

Let us derive the critical points of (2.1).

### Proposition 2.1

The Euler–Lagrange equation of the functional (2.1) is given by

2.3 $$\begin{aligned} \tau (\phi )=Z(\mathrm{d}\phi (e_1)\wedge \mathrm{d}\phi (e_2))+R\nabla V(\phi ), \end{aligned}$$

where $\tau (\phi )$ denotes the tension field of the map $\phi$ and the vector-bundle homomorphism $Z \in \Gamma ({\text {Hom}}(\Lambda ^2T^*N,TN))$ is defined by the equation

2.4 $$\begin{aligned} \Omega (\eta ,\xi _1,\xi _2)=\langle Z(\xi _1\wedge \xi _2),\eta \rangle , \end{aligned}$$

where $\Omega =\mathrm{d}B$ is a three-form on *N* and $e_1,e_2$ an orthonormal basis of *TM*.

### Proof

We consider a smooth variation of $\phi$ that is $\phi _t:(-\epsilon ,\epsilon )\times M\rightarrow N$ with $\frac{\partial \phi _t}{\partial t}\big |_{t=0}=\eta$. It is well-known that

$$\begin{aligned} \frac{\mathrm{{d}}}{\mathrm{{d}}t}\big |_{t=0}\frac{1}{2}\int _M|{\mathrm{d}}\phi _t|^2\mathrm{{d}}M= & {} -\int _M\langle \tau (\phi ),\eta \rangle \mathrm{{d}}M, \\ \frac{\mathrm{{d}}}{\mathrm{{d}}t}\big |_{t=0}\int _M RV(\phi _t)\mathrm{{d}}M= & {} \int _M R\langle \nabla V(\phi ),\eta \rangle \mathrm{{d}}M. \end{aligned}$$

To calculate the variation of the two-form *B*, we choose an orthonormal basis $e_1,e_2$ of *TM* and calculate

$$\begin{aligned} \frac{{\mathrm{d}}}{\mathrm{d}t}(\phi _t^*B)(e_1,e_2)&=\phi _t^*({\mathcal {L}}_{\partial _t}B)(e_1,e_2) \\&=\phi _t^*\big (\iota _{\partial _t}\mathrm{d}B(e_1,e_2)+d(\iota _{\partial _t} B(e_1,e_2))\big ) \\&=\phi _t^*\big (\Omega (\partial _t,e_1,e_2))+d(\phi _t^*(\iota _{\partial _t}B(e_1,e_2))), \end{aligned}$$

where we used that $\mathrm{d}B=\Omega$. Here, ${\mathcal {L}}$ denotes the Lie-derivative applied to differential forms. Moreover, we applied several identities for derivatives of differential forms, which can be found in [24, pp. 170–174].

Integrating over *M* we thus obtain

$$\begin{aligned} \frac{{\mathrm{d}}}{\mathrm{d}t}\big |_{t=0}\int _M(\phi ^*_tB)(e_1,e_2){\mathrm{d}}M=\int _M\Omega (\eta ,{\mathrm{d}}\phi (e_1),{\mathrm{d}}\phi (e_2)){\mathrm{d}}M. \end{aligned}$$

Using the vector-bundle homomorphism (2.4), we thus find

$$\begin{aligned} \frac{{\mathrm{d}}}{\mathrm{d}t}\big |_{t=0}E(\phi _t)=\int _M\langle \eta ,-\tau (\phi )+Z(\mathrm{d}\phi (e_1)\wedge \mathrm{d}\phi (e_2))+R\nabla V(\phi )\rangle {\mathrm{d}}M \end{aligned}$$

yielding the claim. $\square$

We call solutions of (2.3) *harmonic maps with scalar and two-form potential*.

### Remark 2.2

Note that the energy functionals (2.1) and (2.2) have the same critical points since

$$\begin{aligned} \frac{{\mathrm{d}}}{\mathrm{d}t}\big |_{t=0}\exp (iE(\phi _t))=0\Leftrightarrow \frac{{\mathrm{d}}}{\mathrm{d}t}\big |_{t=0}\int _M\left( \frac{1}{2}|{\mathrm{d}}\phi _t|^2+RV(\phi _t)\right) {\mathrm{d}}M+\frac{\mathrm{d}}{{\mathrm{d}}t}\big |_{t=0}E_B(\phi _t)=0. \end{aligned}$$

Whenever using local coordinates, we will use Greek indices on the domain *M* and Latin indices on the target *N*. Moreover, we will make use of the Einstein summation convention, that is, we sum over repeated indices. In terms of local coordinates $x_\alpha$ on *M* and $y^i$ on *N* the Euler–Lagrange equation reads

2.5 $$\begin{aligned} \Delta \phi ^i=-\Gamma ^i_{jk}\frac{\partial \phi ^j}{\partial x_\alpha }\frac{\partial \phi ^k}{\partial x_\beta }h_{\alpha \beta } +Z^i(\partial _{y^j}\wedge \partial _{y^k})\frac{\partial \phi ^j}{\partial x_1}\frac{\partial \phi ^k}{\partial x_2} +Rg^{ik}\frac{\partial V}{\partial y^k}. \end{aligned}$$

### Remark 2.3

The vector-bundle homomorphism *Z* can also be interpreted as arising from a metric connection with totally antisymmetric torsion. In this case one has

$$\begin{aligned} \nabla ^{Tor}_XY=\nabla ^{LC}_XY+A(X,Y), \end{aligned}$$

where $\nabla ^{LC}$ denotes the Levi–Cevita connection, *X*, *Y* are vector fields and *A*(*X*, *Y*) is a skew-adjoint endomorphism. The endomorphism *A*(*X*, *Y*) satisfies

$$\begin{aligned} \langle A(X,Y),Z\rangle =\Omega (X,Y,Z) \end{aligned}$$

with $\Omega \in \Gamma (\Lambda ^3T^*N)$ similar to (2.4). For more details on metric connections with torsion see [3] and references therein.

### Remark 2.4

In principle one could also study the functional (2.1) for a higher-dimensional domain *M* with $m=\dim M\ge 2$. However, then one needs to pull back an *m*-form from the target leading to an Euler–Lagrange equation with a higher nonlinearity on the right hand side, see [17, Chapter 2], for a detailed analysis.

### Lemma 2.5

(Second variation) Let $\phi :M\rightarrow N$ be a harmonic map with scalar and two-form potential. The second variation of the energy functional (2.1) is given by

2.6 $$\begin{aligned}&\frac{\partial ^2}{\partial t^2}\big |_{t=0}E(\phi _t) =\int _M\big (|\nabla \xi |^2-\langle R^N({\mathrm{d}}\phi ,\xi ){\mathrm{d}}\phi ,\xi \rangle +\langle \xi ,(\nabla _\xi Z)(\mathrm{d}\phi (e_1)\wedge \mathrm{d}\phi (e_2))\rangle \nonumber \\&\quad +\langle \xi ,Z(\nabla \xi (e_1)\wedge {\mathrm{d}}\phi (e_2))\rangle +\langle \xi ,Z({\mathrm{d}}\phi (e_1)\wedge \nabla \xi (e_2))\rangle +R{\text {Hess}}V(\xi ,\xi )\big ){\mathrm{d}}M, \end{aligned}$$

where $$\xi =\frac{\partial \phi _t}{\partial t}\big |_{t=0}$$.

### Proof

This follows by a direct calculation. $\square$

### Example 2.6

Suppose that $\phi$ is an isometric immersion from a closed oriented Riemann surface (*M*, *h*) into an oriented Riemannian three manifold (*N*, *g*). In this case the tension field $\phi$ is related to the mean curvature vector $H(\phi )$ by

$$\begin{aligned} \frac{1}{2}\tau (\phi )=H(\phi ). \end{aligned}$$

Any three-form $\Omega$ on *N* must be a multiple of the volume form ${\text {vol}}_g$, that is

$$\begin{aligned} \Omega =f{\text {vol}}_g \end{aligned}$$

for some smooth function $f:N\rightarrow \mathbb {R}$. Thus, if we denote the unit normal field of $\phi (M)\subset N$ by $\nu$ the Euler–Lagrange equation (2.3) is equivalent to

2.7 $$\begin{aligned} 2H(\phi )=\pm (f\nu )\circ \phi +R\nabla V(\phi ). \end{aligned}$$

The sign in front of the first term on the right hand side depends on whether the map $\phi$ is orientation preserving or not, see [17, Remark 2.7].

In case that the function *f* is constant and $\nabla V\sim \nu$ the equation (2.7) has some similarity with the equation for *linear Weingarten surfaces*. These are surfaces immersed in a three-dimensional manifold satisfying

$$\begin{aligned} aH+bK=c, \end{aligned}$$

where *H* denotes the mean curvature, *K* the Gauss curvature and *a*, *b*, *c* are non-zero real numbers.

## Properties of harmonic maps with scalar and two-form potential

In this section we study several properties of solutions of (2.3).

### Lemma 3.1

Let $\phi \in C^2(M,N)$ be a solution of (2.3). Then the following formulas hold:

3.1 $$\begin{aligned} \Delta \frac{1}{2}|{\mathrm{d}}\phi |^2&=|\nabla {\mathrm{d}}\phi |^2-\langle R^N({\mathrm{d}}\phi (e_\alpha ),{\mathrm{d}}\phi (e_\beta )){\mathrm{d}}\phi (e_\alpha ),{\mathrm{d}}\phi (e_\beta )\rangle +\langle {\mathrm{d}}\phi ({\text {Ric}}^M(e_\alpha )),{\mathrm{d}}\phi (e_\alpha )\rangle \nonumber \\&\quad -\langle Z(\mathrm{d}\phi (e_1)\wedge \mathrm{d}\phi (e_2)),\tau (\phi )\rangle +R{\text {Hess}}V({\mathrm{d}}\phi ,\mathrm{d}\phi ) \end{aligned}$$

and

3.2 $$\begin{aligned} \Delta (V\circ \phi )={\mathrm{d}}V\big (R\nabla V(\phi )+Z(\mathrm{d}\phi (e_1)\wedge \mathrm{d}\phi (e_2))\big )+{\text {Tr}}{\text {Hess}}V({\mathrm{d}}\phi ,\mathrm{d}\phi ), \end{aligned}$$

where ${\text {Ric}}^M$ denotes the Ricci-curvature on *M* and $R^N$ the curvature tensor on *N*.

### Proof

This follows by a direct calculation. $\square$

### Definition 3.2

We define a two-tensor by

3.3 $$\begin{aligned} S_{\alpha \beta }:=\frac{1}{2}h_{\alpha \beta }|{\mathrm{d}}\phi |^2-\langle {\mathrm{d}}\phi (e_\alpha ),{\mathrm{d}}\phi (e_\beta )\rangle +RV(\phi )h_{\alpha \beta }. \end{aligned}$$

The tensor $S_{\alpha \beta }$ is called the *stress-energy tensor*.

### Lemma 3.3

The stress-energy tensor $S_{\alpha \beta }$ is divergence free and symmetric if $\phi$ is a harmonic map with scalar and two-form potential.

### Proof

By a direct calculation we find

$$\begin{aligned} \nabla _{e_\alpha }S_{\alpha \beta }&=-\langle \tau (\phi ),{\mathrm{d}}\phi (e_\beta )\rangle +\langle R\nabla V(\phi ),{\mathrm{d}}\phi (e_\beta )\rangle \\&=-\langle Z(\mathrm{d}\phi (e_1)\wedge \mathrm{d}\phi (e_2)),{\mathrm{d}}\phi (e_\beta )\rangle \\&=-\Omega ({\mathrm{d}}\phi (e_1),{\mathrm{d}}\phi (e_2),{\mathrm{d}}\phi (e_\beta ))=0, \end{aligned}$$

which proves the claim. $\square$

Note that $S_{\alpha \beta }$ is no longer trace-free, which corresponds to the fact that the scalar potential $V(\phi )$ in the action functional does not respect the conformal symmetry.

We can express the stress-energy tensor (3.3) invariantly as

3.4 $$\begin{aligned} S_V(\phi )=e(\phi )h-\phi ^*g+RV(\phi )h \end{aligned}$$

with the energy density $e(\phi )=\frac{1}{2}|{\mathrm{d}}\phi |^2$.

With the help of (3.4) we now derive a monotonicity formula for $e(\phi )+RV(\phi )$. A similar calculation for harmonic maps with scalar potential has been carried out in [20]. For the monotonicity formula for harmonic maps we refer to the book [28], for a general treatment of stress-energy tensors with applications to harmonic maps we refer to [2]. Note that we do not have to assume that *M* is compact to derive the monotonicity formula.

Let $(M,h_0)$ be a complete Riemannian surface with a pole $x_0$. Let *r*(*x*) be the Riemannian distance function relative to the point $x_0$. By $\lambda _i,i=1,2$ we denote the eigenvalues of ${\text {Hess}}_{h_0}(r^2)$.

### Proposition 3.4

(Monotonicity formula) Let $\phi :(M,f^2h_0)\rightarrow (N,g)$ be a harmonic map with scalar and two-form potential, where *M* is a complete Riemannian surface and *N* a Riemannian manifold. Suppose that

3.5 $$\begin{aligned} RV(\phi )> 0, \quad r\frac{\partial \log f}{\partial r}\ge 0, \quad \frac{1}{2}\left( \sum _{i=1}^2\lambda _i-2\lambda _\mathrm{max}\right) \ge \sigma \end{aligned}$$

for a positive constant $\sigma$. Then the following inequality holds

3.6 $$\begin{aligned} \frac{\int _{B_{\rho _1}(x_0)}\big (e(\phi )+RV(\phi )\big )}{\rho _1^\sigma }\le \frac{\int _{B_{\rho _2}(x_0)}\big (e(\phi )+RV(\phi )\big )}{\rho _2^\sigma } \end{aligned}$$

for any $0<\rho _1\le \rho _2$. Here, $B_\rho (x)$ denotes the geodesic ball with radius $\rho$ around the point *x*.

The proof is similar to the proof of Theorem 4.1 in [20].

### Proof

For a symmetric (2, 0)-tensor *S* the following identity holds

3.7 $$\begin{aligned} {\text {div}}(\iota _X S)=({\text {div}}S)(X)+\frac{1}{2}\langle S,{\mathcal {L}}_Xh\rangle . \end{aligned}$$

Here, ${\mathcal {L}}_X$ represents the Lie-derivative with respect to the vector field *X*. Integrating the divergence of (3.4) over the ball $B_\rho (x_0)$ with radius $\rho$ around the point $x_0$, using (3.7) and the fact that the stress-energy-tensor is divergence free, we obtain

$$\begin{aligned} \int _{\partial B_{\rho }(x_0)}S_V(\phi )(X,\nu )=\frac{1}{2}\int _{B_{\rho }(x_0)}\langle S_V(\phi ),{\mathcal {L}}_Xh\rangle . \end{aligned}$$

We choose $X=r\frac{\partial }{\partial r}$ and get

$$\begin{aligned} \frac{1}{2}{\mathcal {L}}_Xh=\frac{1}{2}{\mathcal {L}}_X(f^2h_0)=rf\frac{\partial f}{\partial r}h_0+\frac{1}{2}f^2{\mathcal {L}}_Xh_0, \end{aligned}$$

where we used that the metric on *M* is given by $h=f^2h_0$. Hence, we find

$$\begin{aligned} \frac{1}{2}\langle S_V(\phi ),{\mathcal {L}}_Xh\rangle =2RV(\phi )r\frac{\partial \log f}{\partial r}+\frac{1}{2}f^2\langle S_V(\phi ),{\text {Hess}}_{h_0}(r^2)\rangle \end{aligned}$$

since $$\dim M=2$$.

To estimate the last term on the right hand side, we choose a local basis $e_1,e_2$ on *M* with respect to $h_0$ such that ${\text {Hess}}_{h_0}(r^2)$ becomes a diagonal matrix with respect to $e_1,e_2$. Then $\tilde{e}_\alpha :=f^{-1}e_\alpha ,\alpha =1,2$ is an orthonormal basis with respect to *h*. Thus, we obtain

$$\begin{aligned}&f^2\langle S_V(\phi ),{\text {Hess}}_{h_0}(r^2)\rangle =f^2\sum _{\alpha ,\beta =1}^2S_V(\phi )(\tilde{e}_\alpha ,\tilde{e}_\beta ){\text {Hess}}_{h_0}(r^2)(\tilde{e}_\alpha ,\tilde{e}_\beta ) \\&\quad =\sum _{\alpha ,\beta =1}^2\big (e(\phi )h(\tilde{e}_\alpha ,\tilde{e}_\beta )-\phi ^*g(\tilde{e}_\alpha ,\tilde{e}_\beta )+RV(\phi )g(\tilde{e}_\alpha ,\tilde{e}_\beta )\big ){\text {Hess}}_{h_0}(r^2)(e_\alpha ,e_\beta ) \\&\quad =\sum _{\alpha =1}^2(e(\phi )+RV(\phi )){\text {Hess}}_{h_0}(r^2)(e_\alpha ,e_\alpha ) -\sum _{\alpha =1}^2\phi ^*g(\tilde{e_\alpha },\tilde{e_\alpha }){\text {Hess}}_{h_0}(r^2)(e_\alpha ,e_\alpha ) \\&\quad \ge \big (e(\phi )+RV(\phi )\big )\sum _{i=1}^2\lambda _i-2e(\phi )\lambda _\mathrm{max} \\&\quad \ge \big (e(\phi )+RV(\phi )\big )\big (\Lambda -2\lambda _\mathrm{max}\big ), \end{aligned}$$

where $\Lambda =\lambda _1+\lambda _2$. Hence, we obtain

$$\begin{aligned} \frac{1}{2}\langle S_V(\phi ),{\mathcal {L}}_xh\rangle \ge 2RV(\phi )r\frac{\partial \log f}{\partial r}+\frac{1}{2}\big (e(\phi )+RV(\phi )\big )\big (\Lambda -2\lambda _\mathrm{max}\big ). \end{aligned}$$

On the other hand, by the coarea formula and $|\nabla r|=f^{-1}$ we find

$$\begin{aligned} \int _{\partial B_{\rho }(x_0)}S_V(\phi )(r\frac{\partial }{\partial r},\nu )&\le \int _{\partial B_{\rho }(x_0)}(e(\phi )+RV(\phi ))h(r\frac{\partial }{\partial r},\nu )\\&=\rho \int _{\partial B_{\rho }(x_0)}(e(\phi )+RV(\phi ))f\\&=\rho \frac{\mathrm{d}}{\mathrm{d}\rho }\int _{B_\rho (x_0)}\left( \int _{\partial B_{r}(x_0)}\frac{e(\phi )+RV(\phi )}{|\nabla r|}\right) \mathrm{d}r \\&=\rho \frac{\mathrm{d}}{\mathrm{d}\rho }\int _{B_\rho (x_0)}\big (e(\phi )+RV(\phi )\big ). \end{aligned}$$

Thus, by the assumptions (3.5) we obtain the following inequality

$$\begin{aligned} \rho \frac{\mathrm{d}}{\mathrm{d}\rho }\int _{B_\rho (x_0)}(e(\phi )+RV(\phi ))\ge \int _{B_\rho (x_0)}\frac{1}{2}\big (e(\phi )+RV(\phi )\big )\big (\Lambda -2\lambda _\mathrm{max}\big ). \end{aligned}$$

Again, by assumption we get

$$\begin{aligned} \rho \frac{\mathrm{d}}{\mathrm{d}\rho }\int _{B_\rho (x_0)}(e(\phi )+RV(\phi ))\ge \sigma \int _{B_\rho (x_0)}(e(\phi )+RV(\phi )). \end{aligned}$$

This can be rewritten as

$$\begin{aligned} \frac{\mathrm{d}}{\mathrm{d}\rho }\frac{\int _{B_\rho (x_0)}(e(\phi )+RV(\phi ))}{\rho ^\sigma }\ge 0 \end{aligned}$$

and the claim follows by integration with respect to $\rho$. $\square$

As for harmonic maps ([25, Theorem 2]) and harmonic maps with potential ([11, Proposition 2]) we can prove a unique continuation theorem for harmonic maps with scalar and two-form potential. To obtain this result we recall the following ([1, p. 248])

### Theorem 3.5

Let *A* be a linear elliptic second-order differential operator defined on a domain *D* of $\mathbb {R}^n$. Let $u=(u^1,\ldots ,u^n)$ be functions in *D* satisfying the inequality

3.8 $$\begin{aligned} |Au^j|\le C\left( \sum _{\alpha ,i}\big |\frac{\partial u^i}{\partial x^\alpha }\big |+\sum _i|u^i|\right) . \end{aligned}$$

If $u=0$ in an open set, then $u=0$ throughout *D*.

Making use of this result we can prove the following

### Proposition 3.6

Let $\phi ,\phi '\in C^2(M,N)$ be two harmonic maps with scalar and two-form potential. Moreover, assume that $V:N\rightarrow \mathbb {R}$ is a $C^{1,1}$ function. If $\phi$ and $\phi '$ are equal on a connected open set *W* of *M* then they coincide on the whole connected component of *M* which contains *W*.

### Proof

Let *U* be a coordinate ball on *M* such that $\phi =\phi '$ in some open subset. By shrinking *U* if necessary we can assume that both $\phi$ and $\phi '$ map *U* into a single coordinate chart in *N*. We write $y^i(x)$ for $y^i(\phi (x))$ and $z^i(x)$ for $z^i(\phi '(x))$. We consider the function $u^i:=y^i-z^i$. Using the local form of the Euler–Lagrange equation (2.5) we find

$$\begin{aligned} \Delta u^i&=-\Gamma ^i_{jk}(y)\frac{\partial y^j}{\partial x_\alpha }\frac{\partial y^k}{\partial x_\beta }h_{\alpha \beta } +\Gamma ^i_{jk}(z)\frac{\partial z^j}{\partial x_\alpha }\frac{\partial z^k}{\partial x_\beta }h_{\alpha \beta } \\&\quad -Z^i(y)(\partial _{y^j}\wedge \partial _{y^k})\frac{\partial y^j}{\partial x_1}\frac{\partial y^k}{\partial x_2} +Z^i(z)(\partial _{y^j}\wedge \partial _{y^k})\frac{\partial z^j}{\partial x_1}\frac{\partial z^k}{\partial x_2} \\&\quad +Rg^{ik}(y)\frac{\partial V}{\partial y^k}(y)-Rg^{ik}(z)\frac{\partial V}{\partial y^k}(z). \end{aligned}$$

By adding several zero’s on the right hand side the above equation acquires the form (3.8) and the result follows by application of Theorem 3.5. For more details see the proof of Theorem 2 in [25]. $\square$

### Remark 3.7

In the case that the scalar potential $V(\phi )$ vanishes identically, the energy functional (2.1) is conformally invariant. In this case one can exploit the conformal invariance to prove that critical points cannot have isolated singularities, whenever a certain energy is finite. This follows from the main theorem in [14], where a removable singularity theorem for critical points of a large class of conformally invariant energy functionals is established.

As a next step, we use the embedding theorem of Nash to isometrically embed *N* into some $\mathbb {R}^q$. We denote the isometric embedding by $\iota$ and consider the composite map $u:=\iota \circ \phi :M\rightarrow \mathbb {R}^q$. By $\tilde{N}$ we denote the tubular neighborhood of $\iota (N)\subset \mathbb {R}^q$. Let $\pi :\tilde{N}\rightarrow \iota (N)$ be the canonical projection which assigns to each $z\in \tilde{N}$ the closest point in $\iota (N)$ from *z*.

### Lemma 3.8

Assume that $N\subset \mathbb {R}^q$. Then the Euler–Lagrange equation (2.3) acquires the form

3.9 $$\begin{aligned} \Delta u=\mathrm {I\!I}(\mathrm{d}u,\mathrm{d}u)+\tilde{Z}(\mathrm{d}u(e_1)\wedge \mathrm{d}u(e_2))+R\widetilde{\nabla V}(u), \end{aligned}$$

where $\mathrm {I\!I}(\mathrm{d}u,\mathrm{d}u):={\text {Tr}}\nabla \mathrm{d}\pi (\mathrm{d}u,\mathrm{d}u)$. Moreover, $\tilde{Z}$ and $\widetilde{\nabla V}$ denote the extensions of *Z* and $\nabla V$ to the ambient space $\mathbb {R}^q$.

### Proof

This follows from the chain rule for the tension field of composite maps, that is

$$\begin{aligned} \Delta (\iota \circ \phi )=\mathrm{d}\iota (\tau (\phi ))+{\text {Tr}}\nabla \mathrm{d}\pi ({\mathrm{d}}\phi ,\mathrm{d}\phi ). \end{aligned}$$

The homomorphism *Z* and $\nabla V$ can be extended to the ambient space by projecting to a tubular neighborhood, for more details see [18, p. 463] and [12, p. 557]. $\square$

## Existence results via the heat flow

In this section, we derive an existence result for critical points of (2.1) by the heat-flow method. In order to achieve this result, we will assume that $\Omega$ is exact such that we have a variational structure that enables us to derive the necessary estimates for convergence of the gradient flow. A similar approach for geodesics coupled to a magnetic field was performed in [5].

In order to control the non-linearities arising from the two-form, we will have to restrict to target spaces with negative sectional curvature. A similar idea has been used in [27] for the heat flow of the prescribed mean curvature equation. More precisely, we will use the negative curvature of the target to control the non-linearities arising from the two-form potential.

For a general introduction to harmonic maps and their heat flows see the book [19].

The gradient flow of the functional (2.1) is given by

4.1 $$\begin{aligned} \frac{\partial \phi _t}{\partial t}(x,t)=\big (\tau (\phi _t)-Z(\mathrm{d}\phi _t(e_1)\wedge \mathrm{d}\phi _t(e_2))-R\nabla V(\phi _t)\big )(x,t), \end{aligned}$$

where $\phi _t:M\times [0,T)\rightarrow N$ and initial data $\phi (x,0)=\phi _0(x)$. To establish the short-time existence of (4.1) we use the Nash embedding theorem to isometrically embed *N* into $\mathbb {R}^q$.

### Lemma 4.1

Suppose that *N* is isometrically embedded into $\mathbb {R}^q$. Then (4.1) acquires the form

4.2 $$\begin{aligned} \frac{\partial u}{\partial t}=\Delta u-\mathrm {I\!I}(\mathrm{d}u,\mathrm{d}u)-Z(\mathrm{d}u(e_1)\wedge \mathrm{d}u(e_2))-R\nabla V(u), \end{aligned}$$

where $$u:M\times [0,T)\rightarrow \mathbb {R}^q$$.

### Proof

This follows from Lemma 3.8, we will omit the tildes in order not to blow up the notation. $\square$

As a first step, we establish the existence of a short-time solution.

### Lemma 4.2

For $\phi _0\in C^{2+\alpha }(M,N)$ and $V\in C^{1,1}(N)$ there exists a unique, smooth solution to (4.1) for $t\in [0,T_{max})$.

### Proof

This can be proven using the Banach fixed point theorem, which requires that the potential $V\in C^{1,1}(N)$. For more details, see [19, Chapter 5]. $\square$

To extend the solution beyond $T_\mathrm{max}$ we will make use of the following Bochner formulae:

### Lemma 4.3

Let $\phi _t:M\times [0,T_\mathrm{max})\rightarrow N$ be a smooth solution of (4.1). Then we have for all $t\in [0,T_\mathrm{max})$

4.3 $$\begin{aligned} \frac{\partial }{\partial t}\frac{1}{2}|{\mathrm{d}}\phi _t|^2&=\Delta \frac{1}{2}|{\mathrm{d}}\phi _t|^2 -|\nabla {\mathrm{d}}\phi _t|^2 +\langle R^N({\mathrm{d}}\phi _t(e_\alpha ),{\mathrm{d}}\phi _t(e_\beta )){\mathrm{d}}\phi _t(e_\alpha ),{\mathrm{d}}\phi _t(e_\beta )\rangle \nonumber \\&\quad \; -\langle {\mathrm{d}}\phi _t(Ric^M(e_\alpha )),{\mathrm{d}}\phi _t(e_\alpha )\rangle +\langle Z(\mathrm{d}\phi _t(e_1)\wedge \mathrm{d}\phi _t(e_2)),\tau (\phi _t)\rangle \nonumber \\&\quad \; -R{\text {Hess}}V({\mathrm{d}}\phi _t,\mathrm{d}\phi _t) \end{aligned}$$

and

4.4 $$\begin{aligned} \frac{\partial }{\partial t}\frac{1}{2}|\frac{\partial \phi _t}{\partial t}|^2&= \Delta \frac{1}{2}|\frac{\partial \phi _t}{\partial t}|^2-|\nabla \frac{\partial \phi _t}{\partial t}|^2 +\langle R^N({\mathrm{d}}\phi _t(e_\alpha ),\frac{\partial \phi _t}{\partial t}){\mathrm{d}}\phi _t(e_\alpha ),\frac{\partial \phi _t}{\partial t}\rangle \nonumber \\&\quad -\langle \frac{\nabla }{\partial t}Z(\mathrm{d}\phi _t(e_1)\wedge \mathrm{d}\phi _t(e_2)),\frac{\partial \phi _t}{\partial t}\rangle -R{\text {Hess}}V(\frac{\partial \phi _t}{\partial t},\frac{\partial \phi _t}{\partial t}). \end{aligned}$$

### Proof

This follows from the standard Bochner formulas using

$$\begin{aligned} \langle \nabla Z(\mathrm{d}\phi _t(e_1)\wedge \mathrm{d}\phi _t(e_2)),{\mathrm{d}}\phi _t\rangle =-\langle Z(\mathrm{d}\phi _t(e_1)\wedge \mathrm{d}\phi _t(e_2)),\tau (\phi _t)\rangle . \end{aligned}$$

$$\square $$

The Bochner formulae will be the key-tool to achieve long-time existence and convergence of the evolution equation (4.1). However, we have to distinguish between the cases of a compact and a non-compact target.

### Compact target

For a compact target manifold *N* we will prove the following:

#### Theorem 4.4

Let (*M*, *h*) be a closed Riemannian surface and (*N*, *g*) be a closed, oriented Riemannian manifold with negative sectional curvature. Moreover, suppose that $\Omega$ is exact, $|B|_{L^\infty }<1/2$, $V\in C^{2,1}(N)$ and that the homomorphism *Z* satisfies

$$\begin{aligned} \frac{1}{2}|Z|^2_{L^\infty }\le \kappa _N, \end{aligned}$$

where $\kappa _N$ denotes and upper bound on the sectional curvature on *N*. Then $\phi _t:M\times [0,\infty )\rightarrow N$ subconverges as $t\rightarrow \infty$ to a harmonic map with scalar and two-form potential $\phi _\infty$, which is homotopic to $\phi _0$.

We will divide the proof of Theorem 4.4 into several steps.

#### Lemma 4.5

Let $\phi _t:M\times [0,T_\mathrm{max})\rightarrow N$ be a smooth solution of (4.1) and $V\in C^2(N)$. Then for all $t\in [0,T_\mathrm{max})$ the following inequalities hold:

4.5 $$\begin{aligned} \frac{\partial }{\partial t}\frac{1}{2}|{\mathrm{d}}\phi _t|^2\le&\Delta \frac{1}{2}|{\mathrm{d}}\phi _t|^2 +c_1|{\mathrm{d}}\phi _t|^2+\left( \frac{1}{2}|Z|_{L^\infty }^2-\kappa _N\right) |{\mathrm{d}}\phi _t|^4 \end{aligned}$$

and

4.6 $$\begin{aligned} \frac{\partial }{\partial t}\frac{1}{2}|\frac{\partial \phi _t}{\partial t}|^2\le \Delta \frac{1}{2}|\frac{\partial \phi _t}{\partial t}|^2 +\left( |\nabla Z|_{L^\infty }+\frac{1}{4}|Z|_{L^\infty }^2-\kappa _N\right) |{\mathrm{d}}\phi _t|^2|\frac{\partial \phi _t}{\partial t}|^2+c_2|\frac{\partial \phi _t}{\partial t}|^2 \end{aligned}$$

with $c_1:=|{\text {Ric}}|_{L^\infty }+|R|_{L^\infty }|{\text {Hess}}V|_{L^\infty }, c_2:=|R|_{L^\infty }|{\text {Hess}}V|_{L^\infty }$ and $\kappa _N$ denotes an upper bound on the sectional curvature on *N*.

#### Proof

For the first assertion we consider (4.3) and estimate

$$\begin{aligned} |\langle Z(\mathrm{d}\phi _t(e_1)\wedge \mathrm{d}\phi _t(e_2)),\tau (\phi _t)\rangle |\le \sqrt{2}|Z|_{L^\infty }|{\mathrm{d}}\phi _t|^2|\nabla {\mathrm{d}}\phi _t|, \end{aligned}$$

which yields

$$\begin{aligned} \langle Z(\mathrm{d}\phi _t(e_1)\wedge \mathrm{d}\phi _t(e_2)),\tau (\phi _t)\rangle -|\nabla {\mathrm{d}}\phi _t|^2\le \frac{1}{2}|Z|^2_{L^\infty }|{\mathrm{d}}\phi _t|^4. \end{aligned}$$

By assumption *N* is compact and we can estimate the Hessian of the potential $V(\phi )$ by its maximum yielding the first claim.

For the second claim we consider (4.4) and calculate

$$\begin{aligned} \frac{\nabla }{\partial t}Z(\mathrm{d}\phi _t(e_1)\wedge \mathrm{d}\phi _t(e_2))&=(\nabla _{{\mathrm{d}}\phi _t(\partial _t)}Z)(\mathrm{d}\phi _t(e_1)\wedge \mathrm{d}\phi _t(e_2))+Z\left( \frac{\nabla }{\partial t}{\mathrm{d}}\phi _t(e_1)\wedge {\mathrm{d}}\phi _t(e_2)\right) \\&\quad +Z\left( {\mathrm{d}}\phi _t(e_1)\wedge \frac{\nabla }{\partial t}{\mathrm{d}}\phi _t(e_2)\right) , \end{aligned}$$

which gives

$$\begin{aligned} \left\langle \frac{\nabla }{\partial t}Z(\mathrm{d}\phi _t(e_1)\wedge \mathrm{d}\phi _t(e_2)),\frac{\partial \phi _t}{\partial t}\right\rangle&\le |\nabla Z|_{L^\infty }|{\mathrm{d}}\phi _t|^2|\frac{\partial \phi _t}{\partial t}|^2+|Z|_{L^\infty }|\frac{\nabla }{\partial t}{\mathrm{d}}\phi _t||\mathrm{d}\phi _t||\frac{\partial \phi _t}{\partial t}|. \end{aligned}$$

The result follows by applying Young’s inequality to the last term on the right hand side. $\square$

Via the maximum principle we thus obtain the following:

#### Corollary 4.6

Let $\phi _t:M\times [0,T_\mathrm{max})\rightarrow N$ be a smooth solution of (4.1). If $\frac{1}{2}|Z|_{L^\infty }^2\le \kappa ^N$ then for all $t\in [0,T_\mathrm{max})$ the following estimates hold:

4.7 $$\begin{aligned} |{\mathrm{d}}\phi _t|^2&\le |{\mathrm{d}}\phi _0|^2e^{2c_1t}, \end{aligned}$$

4.8 $$\begin{aligned} |\frac{\partial \phi _t}{\partial t}|^2&\le |\frac{\partial \phi _0}{\partial t}|^2e^{\frac{|\nabla Z|_{L^\infty }|{\mathrm{d}}\phi _0|^2}{c_1}e^{2c_1t}+c_2t}. \end{aligned}$$

#### Proof

Using the assumptions the first statement follows by applying the maximum principle to (4.5). For the second statement, we apply the maximum principle to (4.6) using the bound (4.7). $\square$

#### Lemma 4.7

Let $u:M\times [0,T_\mathrm{max})\rightarrow \mathbb {R}^q$ be a smooth solution of (4.2). If $\frac{1}{2}|Z|_{L^\infty }^2\le \kappa ^N$ then the following inequality holds

$$\begin{aligned} |u(\cdot ,t)|_{C^{2+\alpha }(M,N)}+\big |\frac{\partial u}{\partial t}(\cdot ,t)\big |_{C^{\alpha }(M,N)}\le C, \end{aligned}$$

where the constant *C* depends on $M,N,Z,\nabla Z,V,|{\mathrm{d}}\phi _0|$ and $T_\mathrm{max}$.

#### Proof

Interpreting (4.2) as an elliptic equation we may apply elliptic Schauder theory ([16, p. 79]) and get that $u\in C^{1+\alpha }(M,N)$ by the bounds (4.7) and (4.8). Making use of the regularity gained from elliptic Schauder theory we interpret (4.2) as a parabolic equation. The result then follows by application of parabolic Schauder theory ([16, p. 79]). $\square$

#### Lemma 4.8

Let $u,v:M\times [0,T_\mathrm{max})\rightarrow \mathbb {R}^q$ be smooth solutions of (4.2). If $\frac{1}{2}|Z|_{L^\infty }^2\le \kappa ^N$ then the following inequality holds for all $t\in [0,T_\mathrm{max})$

$$\begin{aligned} |u_t-v_t|^2\le |u_0-v_0|^2e^{Ct} \end{aligned}$$

for some positive constant *C*. In particular, if $u_0=v_0$ then $u_t=v_t$ for all $t\in [0,T_\mathrm{max})$.

#### Proof

In the following *C* will denote a universal constant that may change from line to line. We set $h:=\frac{1}{2}|u-v|^2$. By projecting to a tubular neighborhood $\mathrm {I\!I}(\mathrm{d}u,\mathrm{d}u), Z(\mathrm{d}u(e_1)\wedge \mathrm{d}u(e_2))$ and $\nabla V(u)$ can be thought of as vector-valued functions in $\mathbb {R}^q$, for more details see [21, p. 132] and also [12, p. 557]. Exploiting this fact a direct computation yields

$$\begin{aligned} \frac{\partial h}{\partial t}&=\Delta h-|\mathrm{d}h|^2-\langle \mathrm {I\!I}_u(\mathrm{d}u,\mathrm{d}u)-\mathrm {I\!I}_v(\mathrm{d}v,\mathrm{d}v),h\rangle \\&\quad -\langle Z_u(\mathrm{d}u(e_1)\wedge \mathrm{d}u(e_2))-Z_v(\mathrm{d}v(e_1)\wedge \mathrm{d}v(e_2)),h\rangle -R\langle \nabla V(u)-\nabla V(v),h\rangle . \end{aligned}$$

Rewriting

$$\begin{aligned} \mathrm {I\!I}_u(\mathrm{d}u,\mathrm{d}u)-\mathrm {I\!I}_v(\mathrm{d}v,\mathrm{d}v)=(\mathrm {I\!I}_u-\mathrm {I\!I}_v)(\mathrm{d}u,\mathrm{d}u)+\mathrm {I\!I}_v(\mathrm{d}u-\mathrm{d}v,\mathrm{d}u)+\mathrm {I\!I}_v(\mathrm{d}v,\mathrm{d}u-\mathrm{d}v) \end{aligned}$$

and applying the bounds (4.7), (4.8) we find

$$\begin{aligned} |\langle \mathrm {I\!I}_u(\mathrm{d}u,\mathrm{d}u)&-\mathrm {I\!I}_v(\mathrm{d}v,\mathrm{d}v),u-v\rangle |\le C(|u-v|^2+|\mathrm{d}u-\mathrm{d}v||u-v|), \\ |\langle Z_u(\mathrm{d}u(e_1)\wedge \mathrm{d}u(e_2))&-Z_v(\mathrm{d}v(e_1)\wedge \mathrm{d}v(e_2)),u-v\rangle |\le C(|u-v|^2+|\mathrm{d}u-\mathrm{d}v||u-v|) \end{aligned}$$

and the second inequality follows similarly. Moreover, by assumption $V\in C^{2,1}(N)$ and thus

$$\begin{aligned} |\langle \nabla V(u)-\nabla V(v), u-v\rangle |\le Ch. \end{aligned}$$

Consequently, by Young’s inequality we get

$$\begin{aligned} \frac{\partial h}{\partial t}\le \Delta h+Ch \end{aligned}$$

for some positive constant *C*. The result then follows by application of the maximum principle. $\square$

#### Proposition 4.9

Let $\phi _t:M\times [0,T_\mathrm{max})\rightarrow N$ be a smooth solution of (4.1). If $\frac{1}{2}|Z|_{L^\infty }^2\le \kappa ^N$ then there exists a unique smooth solution for all $t\in [0,\infty )$.

#### Proof

This follows from the continuation principle for parabolic partial differential equations. Suppose that there would be a maximal time of existence $T_{fin}$, then using the estimates (4.7) and (4.8) one can show that we can continue the solution for some small $\delta >0$ up to $T_{fin}+\delta$ yielding a contradiction. The uniqueness follows from Lemma 4.8. $\square$

To achieve convergence of the evolution equation (4.1) we will make use of the following:

#### Lemma 4.10

Assume that (*M*, *h*) is a compact Riemannian manifold. If a function $u(x,t)\ge 0$ satisfies

$$\begin{aligned} \frac{\partial u}{\partial t}\le \Delta u+Cu \end{aligned}$$

and if in addition we have the bound

$$\begin{aligned} U(t)=\int _Mu(x,t){\mathrm{d}}M\le U_0, \end{aligned}$$

then there exists a uniform bound on

$$\begin{aligned} u(x,t)\le e^CKU_0 \end{aligned}$$

with the constant *K* depending on *M*.

#### Proof

A proof can for example be found in [26, p. 284]. For more details on how the constant *C* in the estimate depends on geometric data, see [16, Lemma 2.3.1]. $\square$

#### Proposition 4.11

(Convergence) Let $\phi _t:M\times [0,\infty )\rightarrow N$ be a smooth solution of (4.1). If $\Omega$ is exact, $\frac{1}{2}|Z|_{L^\infty }^2\le \kappa ^N$ and $|B|_{L^\infty }<1/2$ then the evolution equation (4.1) subconverges in $C^2(M,N)$ to a harmonic map with scalar and two-form potential $\phi _\infty$, which is homotopic to $\phi _0$.

#### Proof

Since (4.1) is the negative $L^2$ gradient flow of the functional (2.1) we obtain

$$\begin{aligned} \int _M\left( \frac{1}{2}|{\mathrm{d}}\phi _t|^2+\phi _t^*B+RV(\phi _t)\right) {\mathrm{d}}M+\int _0^\infty \int _M\big |\frac{\partial \phi _t}{\partial t}\big |^2{\mathrm{d}}M\mathrm{d}t=E(\phi _0). \end{aligned}$$

Thus, for $|B|_{L^\infty }<1/2$ we get

4.9 $$\begin{aligned} \int _M|{\mathrm{d}}\phi _t|^2{\mathrm{d}}M+\int _0^\infty \int _M\big |\frac{\partial \phi _t}{\partial t}\big |^2{\mathrm{d}}M\mathrm{d}t\le C, \end{aligned}$$

where the constant *C* depends on $|B|_{L^\infty },|R|_{L^\infty }|V|_{L^\infty }$ and $E(\phi _0)$. Applying Lemma 4.10 to (4.5) and (4.9), we thus obtain a uniform bound on $|{\mathrm{d}}\phi _t|^2$. Inserting this bound into (4.6) we find (for some positive constant *C*)

$$\begin{aligned} \frac{\partial }{\partial t}\frac{1}{2}\big |\frac{\partial \phi _t}{\partial t}\big |^2\le \Delta \frac{1}{2}\big |\frac{\partial \phi _t}{\partial t}\big |^2 +C\big |\frac{\partial \phi _t}{\partial t}\big |^2. \end{aligned}$$

Integrating this equation over *M* and *t* from 0 to $\infty$ we get

4.10 $$\begin{aligned} \int _M\big |\frac{\partial \phi _t}{\partial t}\big |^2{\mathrm{d}}M\le C\int _0^\infty \int _M\big |\frac{\partial \phi _t}{\partial t}\big |^2{\mathrm{d}}M\mathrm{d}t +\int _M\big |\frac{\partial \phi _t}{\partial t}\big |^2\big |_{t=0}{\mathrm{d}}M. \end{aligned}$$

At this point we may again apply Lemma 4.10 to (4.6) and (4.10), which gives

$$\begin{aligned} \big |\frac{\partial \phi _t}{\partial t}\big |^2_{L^\infty (M\times [0,\infty ))}\le C. \end{aligned}$$

Now, by (4.9), there exists a sequence $t_k$ such that

$$\begin{aligned} \int _M\big |\frac{\partial \phi _t}{\partial t}(\cdot ,t_k)\big |^2{\mathrm{d}}M\rightarrow 0 \end{aligned}$$

as $k\rightarrow \infty$. Moreover, by Lemma 4.7 we have

$$\begin{aligned} \sup _{k\in \mathbb {N}}\left( \big |\frac{\partial \phi _t}{\partial t}(\cdot ,t_k)\big |_{C^\alpha (M,N)}+|\phi _{t_k}|_{C^{2+\alpha }(M,N)}\right) \le C. \end{aligned}$$

Then it follows by the Theorem of Arzela and Ascoli that there exists a convergent subsequence, which we will also denote by $t_k$. Thus, the family $\phi _{t_k}$ subconverges in $C^2(M,N)$ to a map $\phi _\infty$. Since $\phi _t$ depends smoothly on *t*, the limit $\phi _\infty$ is homotopic to $\phi _0$. $\square$

#### Remark 4.12

Note that we only made use of the globally defined energy (2.1) to obtain convergence of the gradient flow. To achieve long-time existence of (4.1) we do not require a variational structure.

### Non-compact target

In the case that the target manifold *N* is complete, but non-compact we have to make additional assumptions to control the image of *M* under the evolution of $\phi$. However, we can use the potential $V(\phi )$ to constrain $\phi _t(M)$ to a compact set in *N*. This is similar to the case of the heat flow for harmonic maps with potential [12]. To this end, let $\mathrm{d}_N(y)$ denote the Riemannian distance in *N* from some fixed point $y_0$.

Finally, we will prove the following:

#### Theorem 4.13

Let (*M*, *h*) be a closed Riemann surface and (*N*, *g*) a complete, oriented Riemannian manifold with negative sectional curvature. Moreover, suppose that $\Omega$ is exact, $|B|_{L^\infty }<1/2$, $V\in C^{2,1}(N)$ and $\frac{1}{2}|Z|_{L^\infty }^2\le \kappa _N$, where $\kappa _N$ denotes and upper bound on the sectional curvature on *N*. In addition, assume that the potential $V(\phi )$ satisfies

$$\begin{aligned} -R{\text {Hess}}V(y)\le -\frac{C}{1+\mathrm{d}_N(y)} \quad \text { or }\quad |R{\text {Hess}}V|_{L^\infty }\le \frac{\lambda _1(M)}{2}, \end{aligned}$$

where $\lambda _1(M)$ denotes the first eigenvalue of the Laplacian on *M*. Let $\phi _t:M\times [0,\infty )\rightarrow N$ be a smooth solution of (4.1). Then $\phi _t$ subconverges in $C^2(M,N)$ to a harmonic map with scalar and two-form potential, which is homotopic to $\phi _0$. Moreover, $\phi _\infty$ is minimizing the energy in its homotopy class.

First of all, we make the following observation:

#### Lemma 4.14

If the three-form $\Omega$ is exact, then $Z \in \Gamma ({\text {Hom}}(\Lambda ^2T^*N,TN))$ defined via (2.4) is parallel, that is $\nabla Z=0$.

#### Proof

We fix a point $p\in N$ and we extend any tangent vectors $\xi _1,\xi _2,\eta \in T_pN$ to vector fields being *synchronous* in the point *p*, meaning that

$$\begin{aligned} \nabla _V\xi _1=\nabla _V\xi _2=\nabla _V\eta =0 \end{aligned}$$

for all tangent vectors $V\in T_pN$. Then using that $\Omega =\mathrm{d}B$ is exact, we find from (2.4)

$$\begin{aligned} \mathrm{d}B(\xi _1,\xi _2,\eta )=\langle Z(\xi _1\wedge \xi _2),\eta \rangle . \end{aligned}$$

By differentiation we then obtain

$$\begin{aligned} 0=\langle \nabla Z(\xi _1\wedge \xi _2),\eta \rangle , \end{aligned}$$

which proves the claim. $\square$

As a next step we show how we can use the Hessian of the scalar potential $V(\phi )$ to constrain $\phi _t(M)$ to a compact set. This idea has already been applied in the study of the heat flow for harmonic maps with potential, see [12, Proposition 2].

#### Lemma 4.15

Let (*M*, *h*) be a closed Riemann surface and (*N*, *g*) a complete Riemannian manifold. Suppose that $\Omega$ is exact and that $\frac{1}{2}|Z|_{L^\infty }^2\le \kappa _N$. Moreover, assume that the potential $V(\phi )$ satisfies

4.11 $$\begin{aligned} -R{\text {Hess}}V(y)\le -\frac{C}{1+\mathrm{d}_N(y)}. \end{aligned}$$

Then there exists a compact set $K\subset N$ such that $\phi _t(M)\subset K$ as long as the solution of (4.1) exists.

#### Proof

Let $\mathrm{d}_N$ be the distance function from some point $y_0$ in *N*. We set

$$\begin{aligned} f(t):=\sup _{x\in M}\big |\frac{\partial \phi _t}{\partial t}\big |. \end{aligned}$$

It follows directly that

$$\begin{aligned} \mathrm{d}_N(\phi _t)\le C+\int _0^tf(s)\mathrm{d}s. \end{aligned}$$

Making use of the assumptions on the Hessian of the potential (4.11), the homomorphism *Z* and the sectional curvature $K^N$, we obtain from (4.6) by an argument similar to the compact case that

$$\begin{aligned} \frac{\partial }{\partial t}\big |\frac{\partial \phi _t}{\partial t}\big |^2\le \Delta \big |\frac{\partial \phi _t}{\partial t}\big |^2 -\frac{C}{1+\int _0^tf(s)\mathrm{d}s}\big |\frac{\partial \phi _t}{\partial t}\big |^2. \end{aligned}$$

Then, by the maximum principle, we find

$$\begin{aligned} f(t)\le f(0)\exp \left( -\frac{C}{2}\int _0^t\frac{1}{1+\int _0^\tau f(s)\mathrm{d}s}\mathrm{d}\tau \right) . \end{aligned}$$

We can deduce that

$$\begin{aligned} \int _0^tf(s)\mathrm{d}s\le C, \end{aligned}$$

which gives

$$\begin{aligned} f(t)\le f(0)e^{-Ct}. \end{aligned}$$

Hence, we find that $\mathrm{d}_N(y)\le C$. This proves that there exists a compact set $K\subset N$ such that $\phi _t(M)\subset K$ for all $t\in [0,T_\mathrm{max})$. $\square$

As noted in [9, Proposition 2.4], one can also constrain $\phi _t(M)$ to a compact set if the maximum of the Hessian of the potential $V(\phi )$ is bounded by the first eigenvalue of the Laplacian on *M*. This idea can also be applied here:

#### Lemma 4.16

Let (*M*, *h*) be a closed Riemann surface and (*N*, *g*) a complete, oriented Riemannian manifold. Suppose that $\Omega$ is exact and that $\frac{1}{2}|Z|_{L^\infty }^2\le \kappa _N$. Moreover, assume that the potential $V(\phi )$ satisfies

4.12 $$\begin{aligned} |R{\text {Hess}}V|_{L^\infty }\le \frac{\lambda _1(M)}{2}, \end{aligned}$$

where $\lambda _1(M)$ denotes the first eigenvalue of the Laplacian on *M*. Then there exists a compact set $K\subset N$ such that $\phi _t(M)\subset K$ as long as the solution of (4.1) exists.

#### Proof

Using (4.4) we derive the following inequality

$$\begin{aligned} \frac{\partial }{\partial t}\frac{1}{2}\int _M\big |\frac{\partial \phi _t}{\partial t}\big |^2{\mathrm{d}}M&\le \int _M\left( -\big |\nabla \frac{\partial \phi _t}{\partial t}\big |^2 -\kappa _N|{\mathrm{d}}\phi _t|^2\big |\frac{\partial \phi _t}{\partial t}\big |^2+ |Z|_{L^\infty }\big |\nabla \frac{\partial \phi _t}{\partial t}\big ||{\mathrm{d}}\phi _t|\big |\frac{\partial \phi _t}{\partial t}\big | \right. \\ \nonumber&\left. \quad -R{\text {Hess}}V\bigg (\frac{\partial \phi _t}{\partial t},\frac{\partial \phi _t}{\partial t}\bigg )\right) {\mathrm{d}}M \\ \nonumber&\le \int _M\left( -\frac{1}{2}|\nabla \frac{\partial \phi _t}{\partial t}\big |^2+|R{\text {Hess}}V|_{L^\infty }\big |\frac{\partial \phi _t}{\partial t}\big |^2\right) {\mathrm{d}}M, \end{aligned}$$

where we used Young’s inequality and the assumptions on the homomorphism *Z* and the sectional curvature $K^N$ in the last step. By the Kato inequality $\big |\nabla f\big |^2\ge \big |\nabla |f|\big |^2$ for a function $f:M\rightarrow \mathbb {R}$ and the Poincaré inequality on *M* we obtain

$$\begin{aligned} \int _M|\nabla f|^2{\mathrm{d}}M\ge \lambda _1(M)\int _M f^2{\mathrm{d}}M. \end{aligned}$$

Applying this inequality we find

$$\begin{aligned} \frac{\partial }{\partial t}\frac{1}{2}\int _M\big |\frac{\partial \phi _t}{\partial t}\big |^2{\mathrm{d}}M\le \left( |R{\text {Hess}}V|_{L^\infty }-\frac{\lambda _1(M)}{2}\right) \int _M\big |\frac{\partial \phi _t}{\partial t}\big |^2{\mathrm{d}}M \end{aligned}$$

yielding

$$\begin{aligned} \int _M\big |\frac{\partial \phi _t}{\partial t}\big |^2{\mathrm{d}}M\le C_0e^{(2|R{\text {Hess}}V|_{L^\infty }-\lambda _1(M))t}. \end{aligned}$$

Using the assumption on $|R{\text {Hess}}V|_{L^\infty }$ and applying Lemma 4.10 to (4.4), we obtain a uniform pointwise bound on $|\frac{\partial \phi _t}{\partial t}|$. By the same argument as in the previous Lemma this yields the claim. $\square$

By making use of the previous Lemmata we thus find

#### Lemma 4.17

Let (*M*, *h*) be a closed Riemann surface and (*N*, *g*) a complete, oriented Riemannian manifold. Moreover, suppose that $\Omega$ is exact, $|B|_{L^\infty }<1/2$, $V\in C^{2,1}(N)$ and $\frac{1}{2}|Z|_{L^\infty }^2\le \kappa _N$. In addition, assume that the potential $V(\phi )$ satisfies

$$\begin{aligned} -R{\text {Hess}}V(y)\le -\frac{C}{1+\mathrm{d}_N(y)} \quad \text { or }\quad |R{\text {Hess}}V|_{L^\infty }\le \frac{\lambda _1(M)}{2}, \end{aligned}$$

where $\lambda _1(M)$ denotes the first eigenvalue of the Laplacian on *M*. Let $\phi _t:M\times [0,\infty )\rightarrow N$ be a smooth solution of (4.1). Then $\phi _t$ converges in $C^2(M,N)$ to a harmonic map with scalar and two-form potential, which is homotopic to $\phi _0$.

#### Proof

By the previous Lemmata we know that $\phi _t(M)$ stays inside a compact set *K*. Thus, by Theorem 4.4 there exists a sequence $t_k$ such that $\phi _{t_k}$ converges to a harmonic map with scalar and two-form potential. Moreover, we have

$$\begin{aligned} \mathrm{d}_N(\phi _t,\phi _\infty )\le \mathrm{d}_N(\phi _t,\phi _{t_k})+\mathrm{d}_N(\phi _{t_k},\phi _\infty ). \end{aligned}$$

We know that

$$\begin{aligned} \mathrm{d}_N(\phi _t,\phi _{t_k})\le \int _{t_k}^t\big |\frac{\partial \phi _s}{\partial s}\big |\mathrm{d}s\le C\int _{t_k}^te^{-Cs}\mathrm{d}s\rightarrow 0 \end{aligned}$$

as both $k,t\rightarrow \infty$, which proves the claim. $\square$

#### Remark 4.18

In the case of the heat flow for harmonic maps with potential to a non-compact target with a concave potential the limit $\phi _\infty$ is trivial, see [12, p. 564]. This statement relies on the Bochner formula (3.2), due to the presence of the two-form potential we cannot draw the same conclusion here.

#### Remark 4.19

If we would only require an upper bound on $R{\text {Hess}}V$ in *N* then this would be enough to establish long-time existence of (4.1). Consequently, to achieve convergence of (4.1) we need the potential $RV(\phi )$ to constrain $\phi _t(M)$ to a compact set.

#### Remark 4.20

Using the extrinsic version of the evolution equation (4.2) we can apply the maximum principle to bound the image of $\phi _t(M)$. This idea was already used for related geometric flows: a similar criterion as below for the heat flow of harmonic maps with potential is given in [12, Proposition 2]. For the heat flow of the prescribed mean curvature equation in $\mathbb {R}^3$ the same idea is used in Proposition 3.2 in [23].

By a direct calculation using (4.2) we obtain

$$\begin{aligned} \frac{\partial }{\partial t}|u|^2&=\Delta |u|^2-2|\mathrm{d}u|^2+\langle u,\mathrm {I\!I}(\mathrm{d}u,\mathrm{d}u)+Z(\mathrm{d}u(e_1)\wedge \mathrm{d}u(e_2))\rangle -R\langle u,\nabla V(u)\rangle \\&\le \Delta |u|^2-2|\mathrm{d}u|^2(1-\frac{|u|}{2}(|\mathrm {I\!I}|_{L^{\infty }}+|Z|_{L^\infty }))-R\langle u,\nabla V(u)\rangle . \end{aligned}$$

Hence, if we can guarantee that

$$\begin{aligned} \frac{|u|}{2}(|\mathrm {I\!I}|_{L^{\infty }}+|Z|_{L^\infty })\le 1, \quad R\langle u,\nabla V(u)\rangle >0 \end{aligned}$$

at $t=0$, then by the maximum principle we get a bound on $|u|^2$ as long as the solution exists. However, the second condition on the potential *V*(*u*) is hard to ensure.

### Minimizing the energy

In this section, we briefly discuss if the limiting map constructed in Theorem 4.13 is minimizing energy in its homotopy class.

#### Lemma 4.21

Under the assumptions of Theorem 4.13 the limiting map $\phi _\infty$ is minimizing energy in its homotopy class.

#### Proof

We use the formula for the second variation (2.6) to show that the limit $\phi _\infty$ obtained in Theorem 4.13 is minimizing the energy in its homotopy class. Let $\phi _1,\phi _2:M\rightarrow N$ be two smooth maps and let $\Phi :M\times [0,1]$ be a geodesic homotopy between $\phi _1$ and $\phi _2$, that is $\Phi (\cdot ,0)=\phi _1$ and $\Phi (\cdot ,1)=\phi _2$. Moreover, assume that for any $x\in M$ the map $\Phi (x,\cdot )$ is a geodesic, when $\phi _1$ is a harmonic map with scalar and two-form potential. Using the formula for the second variation (2.6), we find the following inequality

$$\begin{aligned} E(\phi _1)-E(\phi _2)&=\int _0^1d\sigma \int _0^\sigma \left( \big |\nabla \frac{\partial \Phi }{\partial s}\big |^2+\kappa _N|{\mathrm{d}}\phi |^2\big |\frac{\partial \Phi }{\partial s}\big |^2 -|Z|_{L^\infty }|{\mathrm{d}}\phi |\big |\nabla \frac{\partial \Phi }{\partial s}\big |\big |\frac{\partial \Phi }{\partial s}\big |\right. \\&\left. \quad -R{\text {Hess}}V\bigg (\frac{\partial \Phi }{\partial s},\frac{\partial \Phi }{\partial s}\bigg ) \right) \mathrm{d}s \\&\ge \int _0^1d\sigma \int _0^\sigma \left( \frac{1}{2}\big |\nabla \frac{\partial \Phi }{\partial s}\big |^2+(\kappa ^N-\frac{1}{2}|Z|^2_{L^\infty })|{\mathrm{d}}\phi |^2\big |\frac{\partial \Phi }{\partial s}\big |^2 \right. \\&\left. \quad -R{\text {Hess}}V\bigg (\frac{\partial \Phi }{\partial s},\frac{\partial \Phi }{\partial s}\bigg )\right) \mathrm{d}s \\&>0. \end{aligned}$$

Thus, under the assumptions of the Lemma $\phi _1$ is an energy minimizer in its homotopy class. $\square$

#### Remark 4.22

Let $\phi _t:M\times [0,\infty )\rightarrow N$ be a smooth solution of (4.1). Under the assumptions of Theorem 4.13 the energy $E(\phi (t))$ is a convex function of *t*, which follows by a direct calculation.

Exploiting the convexity of the energy $E(\phi (t))$ with respect to *t* we can prove the following uniqueness Theorem, which is very similar to the case of Hartman’s theorem for harmonic maps [15].

#### Proposition 4.23

Under the assumptions of Theorem 4.13 the limit $\phi _\infty$ is independent of the chosen subsequence $t_k$.

#### Proof

The proof is the same as in the case of harmonic maps and we omit it here. $\square$

## References

[1] Aronszajn N (1957). A unique continuation theorem for solutions of elliptic partial differential equations or inequalities of second order. J. Math. Pures Appl..

[2] Baird P (2008). Stress-energy tensors and the Lichnerowicz Laplacian. J. Geom. Phys..

[3] Branding, V.: Dirac-harmonic maps with torsion. Commun. Contemp. Math. **18**(4) (2016)

[4] Branding V (2015). Magnetic Dirac-harmonic maps. Anal. Math. Phys..

[5] Branding, V., Hanisch, F. Magnetic geodesics via the heat flow. (2014). arxiv:1411.6848

[6] Chen Q (1998). Harmonic maps with potential from complete manifolds. Chin. Sci. Bull..

[7] Chen Q (1998). Liouville theorem for harmonic maps with potential. Manuscripta Math..

[8] Chen Q (1999). Maximum principles, uniqueness and existence for harmonic maps with potential and Landau–Lifshitz equations. Calc. Var. Partial Differ. Equ..

[9] Chen Q, Zhou Z-R (2003). Heat flows of harmonic maps with potential into manifolds with nonpositive curvature. Arch. Math. (Basel).

[10] Eells J, Sampson JH (1964). Harmonic mappings of Riemannian manifolds. Am. J. Math..

[11] Fardoun A, Ratto A (1997). Harmonic maps with potential. Calc. Var. Partial Differ. Equ..

[12] Fardoun A, Ratto A, Regbaoui R (2000). On the heat flow for harmonic maps with potential. Ann. Global Anal. Geom..

[13] Fuchs, J., Nikolaus, T., Schweigert, C., Waldorf, K.: Bundle gerbes and surface holonomy. In: European Congress of Mathematics, pp. 167–195. Eur. Math. Soc., Zürich (2010)

[14] Grüter M (1984). Conformally invariant variational integrals and the removability of isolated singularities. Manuscripta Math..

[15] Hartman P (1967). On homotopic harmonic maps. Can. J. Math..

[16] Jost, J.: Nonlinear methods in Riemannian and Kählerian geometry. DMV Seminar, vol. 10. Birkhäuser Verlag, Basel (1988)

[17] Koh, D.: The evolution equation for closed magnetic geodesics. Dissertation/PhD-thesis, Universitätsverlag Potsdam (2008)

[18] Koh D (2009). On the evolution equation for magnetic geodesics. Calc. Var. Partial Differ. Equ..

[19] Lin F, Wang C (2008). The Analysis of Harmonic Maps and Their Heat Flows.

[20] Lin H, Yang G, Ren Y, Chong T (2012). Monotonicity formulae and Liouville theorems of harmonic maps with potential. J. Geom. Phys..

[21] Nishikawa, S.: Variational Problems in Geometry, Volume 205 of Translations of Mathematical Monographs. American Mathematical Society, Providence (2002). Translated from the 1998 Japanese original by Kinetsu Abe, Iwanami Series in Modern Mathematics

[22] Polchinski, J.: String Theory. Vol. I. Cambridge Monographs on Mathematical Physics. Cambridge University Press, Cambridge (2005). An introduction to the bosonic string, Reprint of the 2003 edition

[23] Rey O (1991). Heat flow for the equation of surfaces with prescribed mean curvature. Math. Ann..

[24] Rudolph, G., Schmidt, M.: Differential Geometry and Mathematical Physics. Part I. Theoretical and Mathematical Physics. Springer, Dordrecht (2013). Manifolds, Lie groups and Hamiltonian systems

[25] Sampson JH (1978). Some properties and applications of harmonic mappings. Ann. Sci. École Norm. Sup. (4).

[26] Taylor ME (2011). Partial Differential Equations III. Nonlinear Equations, Volume 117 of Applied Mathematical Sciences.

[27] Toda M (1997). On the existence of $$H$$-surfaces into Riemannian manifolds. Calc. Var. Partial Differ. Equ..

[28] Xin Y (1996). Geometry of Harmonic Maps. Progress in Nonlinear Differential Equations and Their Applications.

