# Correction to: The heat flow for the full bosonic string

**DOI:** 10.1007/s10455-017-9591-z

**Published:** 2018-02-12

**Authors:** Volker Branding

**Affiliations:** Faculty of Mathematics, University of Vienna, Oskar-Morgenstern-Platz 1, 1090 Vienna, Austria

## Correction to: Ann Glob Anal Geom (2016) 50:347–365 10.1007/s10455-016-9514-4

As noted by Xuangzhi Cao and Qun Chen the term $|\hbox {d}\phi _t(e_1)\wedge \hbox {d}\phi _t(e_2)|^2$ was written as $|\hbox {d}\phi _t|^4$ at several places in [1], which we will correct below.

Regarding [1, Lemma 4.5], the corrected version looks like:

### Lemma

Let $\phi _t:M\times [0,T_{\max })\rightarrow N$ be a smooth solution of (4.1) in [1] and $V\in C^2(N)$. Assume that $N$ has negative sectional curvature. Then for all $t\in [0,T_{\max })$ the following inequalities hold:

0.1 $$\begin{aligned} \frac{\partial }{\partial t}\frac{1}{2}|\mathrm{d}\phi _t|^2&\le \Delta \frac{1}{2}|\mathrm{d}\phi _t|^2 +c_1|\mathrm{d}\phi _t|^2+\left( \frac{1}{2}|Z|_{L^\infty }^2-2\kappa _N\right) |\mathrm{d}\phi _t(e_1)\wedge \mathrm{d}\phi _t(e_2)|^2 \end{aligned}$$

and

0.2 $$\begin{aligned} \frac{\partial }{\partial t}\frac{1}{2}\left| \frac{\partial \phi _t}{\partial t}\right| ^2&\le \Delta \frac{1}{2}\left| \frac{\partial \phi _t}{\partial t}\right| ^2 +\left( |\nabla Z|_{L^\infty }+\frac{1}{4}|Z|_{L^\infty }^2\right) |\mathrm{d}\phi _t|^2\left| \frac{\partial \phi _t}{\partial t}\right| ^2+c_2\left| \frac{\partial \phi _t}{\partial t}\right| ^2 \end{aligned}$$

with $c_1:=|\mathrm{Ric}^{\mathrm{M}}|_{L^\infty }+|R|_{L^\infty }|{\text {Hess}}V|_{L^\infty }, c_2:=|R|_{L^\infty }|{\text {Hess}}V|_{L^\infty }$ and the positive number $\kappa _N$ denotes an upper bound on the absolute value of the sectional curvature of $N$.

### Proof

We have the following Bochner formula (formula (4.3) in [1])

$$\begin{aligned} \frac{\partial }{\partial t}\frac{1}{2}|\hbox {d}\phi _t|^2&=\Delta \frac{1}{2}|\hbox {d}\phi _t|^2 -|\nabla \hbox {d}\phi _t|^2 +\langle R^N(\hbox {d}\phi _t(e_\alpha ),\hbox {d}\phi _t(e_\beta ))\hbox {d}\phi _t(e_\alpha ),\hbox {d}\phi _t(e_\beta )\rangle \\&\quad -\langle \hbox {d}\phi _t(Ric^M(e_\alpha )),\hbox {d}\phi _t(e_\alpha )\rangle +\langle Z(\mathrm{d}\phi _t(e_1)\wedge \mathrm{d}\phi _t(e_2)),\tau (\phi _t)\rangle \nonumber \\&\quad -R{\text {Hess}}V(\hbox {d}\phi _t,\hbox {d}\phi _t). \end{aligned}$$

First, we estimate

$$\begin{aligned} |\langle Z(\mathrm{d}\phi _t(e_1)\wedge \mathrm{d}\phi _t(e_2)),\tau (\phi _t)\rangle |\le \sqrt{2}|Z|_{L^\infty }|\mathrm{d}\phi _t(e_1)\wedge \mathrm{d}\phi _t(e_2)||\nabla \mathrm{d}\phi _t|, \end{aligned}$$

which yields

$$\begin{aligned} \langle Z(\mathrm{d}\phi _t(e_1)\wedge \mathrm{d}\phi _t(e_2)),\tau (\phi _t)\rangle -|\nabla \mathrm{d}\phi _t|^2\le \frac{1}{2}|Z|^2_{L^\infty }|\mathrm{d}\phi _t(e_1)\wedge \mathrm{d}\phi _t(e_2)|^2. \end{aligned}$$

Furthermore, we find (see also [2, Lemma 3.1])

$$\begin{aligned} \left\langle R^N(\hbox {d}\phi _t(e_\alpha ),\hbox {d}\phi _t(e_\beta ))\hbox {d}\phi _t(e_\alpha ),\hbox {d}\phi _t(e_\beta )\right\rangle&= 2\left\langle R^N(\hbox {d}\phi _t(e_1),\hbox {d}\phi _t(e_2))\hbox {d}\phi _t(e_1),\hbox {d}\phi _t(e_2)\right\rangle \\&=2\left\langle Q^N(\hbox {d}\phi _t(e_1)\wedge \hbox {d}\phi _t(e_2)),\hbox {d}\phi _t(e_1)\wedge \hbox {d}\phi _t(e_2)\right\rangle \\&\le -\,2\kappa ^N|\hbox {d}\phi _t(e_1)\wedge \hbox {d}\phi _t(e_2)|^2.\\ \end{aligned}$$

Here, $Q^N$ denotes the curvature operator on $N$. By assumption $N$ is compact and we can estimate the Hessian of the potential $V(\phi )$ by its maximum yielding the first claim.

Regarding the second statement, we want to mention that the original proof is correct. $\square$

Via the maximum principle we thus obtain the following (which is Corollary 4.6 in [1] with adjusted constants).

### Corollary

If $\frac{1}{2}|Z|_{L^\infty }^2\le \kappa ^N$ then for all $t\in [0,T_{\max })$ the following estimates hold:

0.3 $$\begin{aligned} |\mathrm{d}\phi _t|^2&\le |\mathrm{d}\phi _0|^2e^{2c_1t}, \end{aligned}$$

0.4 $$\begin{aligned} \left| \frac{\partial \phi _t}{\partial t}\right| ^2&\le \left| \frac{\partial \phi _0}{\partial t}\right| ^2e^{\frac{\left( |\nabla Z|_{L^\infty }+\frac{1}{4}|Z|^2_{L^\infty }\right) |\mathrm{d}\phi _0|^2}{c_1}e^{2c_1t}+c_2t}. \end{aligned}$$

We also correct a typo in the proof of [1, Lemma 4.8]. The equation on lines 5 and 6 on page 358 should be

$$\begin{aligned} \frac{\partial h}{\partial t}&=\Delta h-|\hbox {d}u-\hbox {d}v|^2-\langle \mathrm {I\!I}_u(\hbox {d}u,\hbox {d}u)-\mathrm {I\!I}_v(\hbox {d}v,\hbox {d}v),u-v\rangle \\&\quad -\langle Z_u(\hbox {d}u(e_1)\wedge \hbox {d}u(e_2))-Z_v(\hbox {d}v(e_1)\wedge \hbox {d}v(e_2)),u-v\rangle \\&\quad -R\langle \nabla V(u)-\nabla V(v),u-v\rangle . \end{aligned}$$

We also want to point out that one needs the following statement in the proofs of Lemma 4.15 and Lemma 4.16, which was not explicitly given in [1].

### Lemma

Let $\phi _t:M\times [0,T_{\max })\rightarrow N$ be a smooth solution of (4.1) in [1] with $V\in C^2(N)$ and $\nabla Z=0$. Assume that $N$ has negative sectional curvature. If $\frac{1}{2}|Z|_{L^\infty }^2\le \kappa ^N$ then for all $t\in [0,T_{\max })$ the following inequality holds

0.5 $$\begin{aligned} \frac{\partial }{\partial t}\frac{1}{2}\left| \frac{\partial \phi _t}{\partial t}\right| ^2&\le \Delta \frac{1}{2}\left| \frac{\partial \phi _t}{\partial t}\right| ^2-\frac{1}{2}\left| \nabla \frac{\partial \phi _t}{\partial t}\right| ^2-R{\text {Hess}}V\left( \frac{\partial \phi _t}{\partial t},\frac{\partial \phi _t}{\partial t}\right) . \end{aligned}$$

### Proof

Here, we make use of the following Bochner formula ((4.4) in [1])

$$\begin{aligned} \frac{\partial }{\partial t}\frac{1}{2}\left| \frac{\partial \phi _t}{\partial t}\right| ^2&= \Delta \frac{1}{2}\left| \frac{\partial \phi _t}{\partial t}\right| ^2-\left| \nabla \frac{\partial \phi _t}{\partial t}\right| ^2 +\left\langle R^N\left( \mathrm{d}\phi _t(e_\alpha ),\left( \frac{\partial \phi _t}{\partial t}\right) \mathrm{d}\phi _t(e_\alpha )\right) ,\frac{\partial \phi _t}{\partial t}\right\rangle \nonumber \\&\quad -\left\langle \frac{\nabla }{\partial t}Z(\mathrm{d}\phi _t(e_1)\wedge \mathrm{d}\phi _t(e_2)),\frac{\partial \phi _t}{\partial t}\right\rangle -R{\text {Hess}}V\left( \frac{\partial \phi _t}{\partial t},\frac{\partial \phi _t}{\partial t}\right) . \end{aligned}$$

We calculate

$$\begin{aligned} \frac{\nabla }{\partial t}Z(\mathrm{d}\phi _t(e_1)\wedge \mathrm{d}\phi _t(e_2))&=Z\left( \frac{\nabla }{\partial t}\mathrm{d}\phi _t(e_1)\wedge \mathrm{d}\phi _t(e_2)\right) +Z\left( \mathrm{d}\phi _t(e_1)\wedge \frac{\nabla }{\partial t}\mathrm{d}\phi _t(e_2)\right) , \end{aligned}$$

which allows us to estimate

$$\begin{aligned} \left\langle \frac{\nabla }{\partial t}Z(\mathrm{d}\phi _t(e_1)\wedge \mathrm{d}\phi _t(e_2)),\frac{\partial \phi _t}{\partial t}\right\rangle&\le |Z|_{L^\infty }\left| \frac{\nabla }{\partial t}\mathrm{d}\phi _t\right| \left| \mathrm{d}\phi _t\wedge \frac{\partial \phi _t}{\partial t}\right| ,\\ \left\langle R^N(\mathrm{d}\phi _t(e_\alpha ),\left( \frac{\partial \phi _t}{\partial t}\right) \mathrm{d}\phi _t(e_\alpha ),\frac{\partial \phi _t}{\partial t}\right\rangle&\le -\kappa ^N\left| \mathrm{d}\phi _t\wedge \frac{\partial \phi _t}{\partial t}\right| ^2. \end{aligned}$$

The result follows by applying Young’s inequality. $\square$

We also correct several typos in [1, Lemma 4.21]. The inequality given at the bottom should be

$$\begin{aligned} E(\phi _1)-E(\phi _2)&=\int _0^1\hbox {d}\sigma \int _0^\sigma \left( \left| \nabla \frac{\partial \Phi }{\partial s}\right| ^2-\left\langle R^N\left( \hbox {d}\Phi ,\frac{\partial \Phi }{\partial s}\right) \hbox {d}\Phi ,\frac{\partial \Phi }{\partial s}\right\rangle \right. \\&\quad +\left\langle \frac{\partial \Phi }{\partial s},Z\left( \frac{\nabla }{\partial s}\hbox {d}\Phi (e_1)\wedge \hbox {d}\Phi (e_2)\right) \right\rangle \\&\quad \left. +\left\langle \frac{\partial \Phi }{\partial s},Z\left( \hbox {d}\Phi (e_1)\wedge \frac{\nabla }{\partial s}\hbox {d}\Phi (e_2)\right) \right\rangle +R{\text {Hess}}V\left( \frac{\partial \Phi }{\partial s},\frac{\partial \Phi }{\partial s}\right) \right) \hbox {d}s \\&\ge \int _0^1\hbox {d}\sigma \int _0^\sigma \left( \left| \nabla \frac{\partial \Phi }{\partial s}\right| ^2+\kappa _N\left| \hbox {d}\Phi \wedge \frac{\partial \Phi }{\partial s}\right| ^2 -|Z|_{L^\infty }\left| \hbox {d}\Phi \wedge \frac{\partial \Phi }{\partial s}\right| \left| \nabla \frac{\partial \Phi }{\partial s}\right| \right. \\&\left. \quad +R{\text {Hess}}V\left( \frac{\partial \Phi }{\partial s},\frac{\partial \Phi }{\partial s}\right) \right) \hbox {d}s \\&\ge \int _0^1\hbox {d}\sigma \int _0^\sigma \left( \frac{1}{2}\left| \nabla \frac{\partial \Phi }{\partial s}\right| ^2+\left( \kappa ^N-\frac{1}{2}|Z|^2_{L^\infty }\right) \left| \hbox {d}\Phi \wedge \frac{\partial \Phi }{\partial s}\right| ^2 \right. \\&\left. \quad +R{\text {Hess}}V\left( \frac{\partial \Phi }{\partial s},\frac{\partial \Phi }{\partial s}\right) \right) \hbox {d}s \\&>0. \end{aligned}$$
